# Circulating Fibroblast Growth Factor-23 is Associated with Cardiovascular Prognosis and Graft Function in Renal Transplant Recipients

**DOI:** 10.7759/cureus.7140

**Published:** 2020-02-29

**Authors:** Beyza Algul Durak, Mine Sebnem Karakan

**Affiliations:** 1 Nephrology, Ankara City Hospital, Ankara, TUR; 2 Nephrology, Ankara Yildirim Beyazit University, Ankara, TUR

**Keywords:** fibroblast growth factor-23, atherosclerosis, kidney transplant

## Abstract

Purpose

The aim of this study was to evaluate the relationship between Fibroblast Growth Factor-23 (FGF23) serum levels and cardiovascular disease and early graft failure in renal transplant recipients.

Methods

This cross-sectional study was conducted on renal transplant recipients followed by our adult kidney transplant clinic. The patients were divided into two groups according to the mean FGF23 levels (mean FGF23 level=71.2 ± 34.6pg/mL). The patients included in the study were classified as Group 1 (FGF23 <71 pg/mL, n= 42) and Group 2 (FGF23 ≥ 71pg/mL, n= 46) and the data was analyzed as a statistical significance between the two groups. The presence of atherosclerosis was determined by a Doppler ultrasound for evaluate the carotid artery intima-media thickness (CA-IMT). Intrarenal Doppler spectra were obtained with same Doppler ultrasound to determine the renal resistivity index (RRI) for evaluate graft renal failure.

Results

A total of 88 kidney transplantation recipients were included in the study. In the multivariate analysis adjusted for age and gender, the eGFR (β =-0.217, p=0.048), CA-IMT (β =0.318, p=0.009) and RRI (β =0.246, p=0.019) parameters were statistically significant, while the remaining parameters were not statistically significant. In the group analysis, Ca (9.6 ± 0.3 vs. 8.8 ± 0,2, p< 0.05), CA-IMT (0.9 ± 0.2, vs. 0.6 ± 0.3, p< 0.05) and RRI (0.69 ± 0.04 vs. 0.60 ± 0.01, p< 0.05) were significantly higher in the patients in group 2 than the patients in group 1.

Conclusion

According to our results, FGF23 can be considered as a descriptive biomarker for cardiovascular prognosis and graft function for patients with kidney transplantation.

## Introduction

Chronic kidney disease (CKD) is a growing public health problem with a prevalence rate of approximately 8-16% all over the world [1]. End-stage kidney disease (ESRD) requiring renal replacement therapy (RRT) affects more than 2 million people worldwide and kidney transplant is considered to be a better RRT than dialysis [2, 3]. In general, CKD is associated with mineral and bone metabolism disorders arising due to either one or a combination of the following factors: abnormalities of phosphorus, Fibroblast Growth Factor-23 (FGF23), calcium, parathyroid hormone (PTH), and vitamin D metabolism [4]. The majority of kidney disease-mineral and bone disorder (CKD-MBD) factors mostly show up in cases where estimated glomerular filtration rate (eGFR) decreases below 40 mL/min [4]. Recognizable abnormalities in extraskeletal calcification were elevated FGF23 secretion, loss of klotho; reduced rates of bone formation rates may come out earlier in the course of CKD [5]. Fibroblast growth factor-23 (FGF23) is a hormone that is mainly secreted by osteocytes and to a lesser extent by osteoblasts, hypothalamus, endocrine organs, thalamus, and heart. Soluble klotho (s-KL) acts as a co-receptor for FGF23 [6]. An increase in the levels of serum FGF23 in CKD patients are seen from the early stages of the disease [7]. However, poor renal function negatively affects Klotho levels and an increase in FGF23 with Klotho deficiency has been reported to promote vascular calcification and arterial stiffness [8]. In numerous studies conducted previously, it has been reported that increased serum P and intact parathyroid hormone (iPTH) levels, and reduced levels of serum 1,25-dihydroxyvitamin D (1,25(OH)2D) go along with an elevation in serum FGF23 levels in patients with CKD [6, 9].

Cardiovascular diseases (CVD) are the leading cause of morbidity and mortality in CKD patients [10]. Patients with CKD have several risk factors that may predispose them to cardiovascular events, such as chronic inflammatory state of uremia, mineral and bone disorders and anemia, the “classic” cardiovascular risk factors [11]. Since inflammation is the primary cause of CVD in patients with CKD, FGF23 is correlated with an increased risk of developing cardiovascular events and/or death in these population [12-15]. FGF23 all by itself causes atherosclerosis and increased arterial stiffness in rodents, non-uremic subjects and CKD, with a resulting elevation in pulse pressure. In addition, intermediate outcomes such as common Carotid artery intima-media thickness (CA-IMT) measured by ultrasound is among the first arterial wall anomalies that characterise the early phases of plaque formation [11]. There is growing evidence that carotid CA-IMT reflects the severity of atherosclerosis and arterial stiffness, and has been known as independent determinant of cardiovascular events and mortality in these patients [11].

Patients who have undergone kidney transplantation have also been shown to have increased FGF23 levels, even in the case of normal graft function [16]. Kidney transplantation promotes specific alterations in the phosphate metabolism characterized by severe hypophosphataemia (<0.5 mmol/l) related to relatively high levels of parathyroid hormone (PTH) and FGF23 within the first three months of transplantation. This is followed by normalization of these three factors, usually within the first year of transplantation, and a recurrent high FGF23 and PTH levels associated with a decline in graft functions in the late post-transplantation stage [17].

In renal transplant recipients, a good marker for early detection of loss of graft function and early markers of atherosclerosis (an indicator of CVD risk factors) has not yet been found. FGF23 may be an early indicator of both early graft loss and increased cardiovascular risk in these patients. We hypothesized that FGF23 was associated with CVD and early graft failure in renal transplant recipients (RTR).

## Materials and methods

This cross-sectional study was conducted on 88 (54% male, mean age 44.9 ± 8.7 years) renal transplant recipients followed by our adult kidney transplant clinic between February 2018- January 2019. The study exclusion criteria were patients under 18 years old who were determined to have a recent cardiovascular event (within the last month left ventricular ejection fraction of <45%, congestive heart failure, heart valve disease, cardiac pacemaker, arrhythmia), an episode of active infection, a bone fracture in the past year, active malignancy, active vasculitis or diabetes mellitus, Paget's disease, serum creatinine of >2.5 mg/dL, and a renal transplantation duration of <1 year. Until enrollment, none of the participants were treated with folate, vitamin D, vitamin B12, erythropoietin or biphosphonate. The etiologies of pretransplant renal failure were as follows: hypertensive nephrosclerosis (55.68%), chronic diabetes (18.18%), nephrolithiasis (6%), glomerulonephritis (10.22%), polycystic kidney disease (5.6%), and others or unknown etiology (3.4%). All patients were receiving triple therapy (immunosuppressive therapy) including calcineurin inhibitor (CNI) (cyclosporine or tacrolimus) along with mycophenolate mofetil (MMF)/mycophenolate sodium (MNa) and steroids. During transplantation, induction therapy was carried out with basiliximab or anti-thymocyte globulin. The study protocol was approved by the local scientific ethics committee and informed consent was obtained from all patients.

The demographic characteristics, blood pressure values, medications, and smoking history of the patients were recorded. Body mass index (BMI) was calculated as weight in kilograms divided by height in meters squared. Fasting blood sample was taken from each patient. C-reactive protein (CRP), total cholesterol, low-density lipoprotein cholesterol, high-density lipoproteins, serum creatinine, triglycerides, albumin, phosphorus, alkaline phosphatase, calcium, and parathyroid hormone were measured for all participants using standard methods. Serum samples and plasma separated within 30 minutes were stored at -80 °C if not analyzed immediately. The levels of FGF23 (Awarnes, Shanghai, China) were calculated using commercial enzyme-linked immunoadsorbent assay (ELISA) kits. The FGF23 measurement range was determined as 15.63-1000 pg/mL and the measurement sensitivity as 9.38 pg/mL. Meanwhile, eGFR was estimated based on the Modification of Diet in Renal Disease formula [18].

Carotid artery intima-media thickness measurement

The presence of atherosclerosis was determined by a Doppler ultrasound (USG) device using a 2-5 mHz linear probe with (Hitachi Hi-Vision avius, Tokyo, Japan). Imaging of the carotid artery was carried out when all participants were lying in the supine position with their neck slightly extended and head rotated about 20° from the side being examined, and performed after 15 min of rest in a supine position. The measurements were taken from the right-left distal common carotid artery bifurcation, and three points on the distal walls of the proximal portion of the internal carotid artery. Carotid artery intima-media thickness (CA-IMT) was determined by longitudinal examination from the distance defined as the interface between vascular lumen echogenicity and media/adventitia echogenicity [19]. Both right and left CA-IMT measurements were made 3 times, and the average of the 3 measurements was used to determine by experienced investigators. A CA-IMT value of ≥ 0.9 mm was considered as the development of atherosclerosis [20]. Intrarenal Doppler spectra were obtained with same Doppler ultrasound device five regions of segmental arteries. Renal Resistivity Index (RRI) was determined by the same investigators with the following formula: (peak systolic velocity-peak diastolic velocity)/peak systolic velocity.

The patients were divided into two groups according to the mean FGF23 levels (mean FGF23 level=71.2 ± 34.6pg/mL). The patients included in the study were classified as Group 1 (FGF23 <71 pg/mL, n= 42) and Group 2 (FGF23 ≥ 71pg/mL, n= 46) and the data were analyzed as a statistical significance between the two groups.

Statıstıcal Analysis 

Continuous variables were determined as mean ± standard deviation and categorical variables as percentage (%). The Mann-Whitney U test, Student's t test, Pearson chi-square test and Fisher's Exact tests were used to compare the patient characteristics with that of the healthy control group. A p value of <0.05 was considered statistically significant. Statistical analyses were carried out using the SPSS computer software (version 13; SPSS Inc, Chicago, Ill., USA).

## Results

Our study included 88 kidney transplantation recipients. The mean age of the patients was 44.9 ± 8.7 years. Of the study patients, 54% were male. The duration of dialysis before transplantation was 42.3 ± 16.7 months and the mean duration after transplantation was 61.2 ± 22.5 months. The demographic, clinical and laboratory parameters of the study are shown in Table 1. The patients' kidney failure etiology and the immunosuppression protocol used are also shown in Table 1. In the correlation analysis, it was found that there was a statistically significant negative correlation between serum FGF23 and eGFR (r=-0.21, p<0.05) and statistically significant positive correlation between duration of dialysis before transplantation (month) (r=0.22, p<0.05), calcium (r=0.54 , p<0.05), CA-IMT (mm) (r=0.18, p<0.05) and RRI (r=0.30, p<0.05) (Table 2).

**Table 1:** Demographic data and laboratory values of patients

Parameters		Patients (n=88 ), Mean ± SD/(%)
Age (years)		44.9 ± 8.7
Male/Female		%54/%46
Duration of dialysis before transplantation (months)		42.3 ±16.7
Duration after transplantation (months)		61.2 ± 22.5
Use of immunosuppressive drugs	Cyclosporine-MMF/MNa-steroid	20 (22.7%)
	Tacrolimus-MMF/MNa-steroid	68 (77.3%)
Etiology of ESRD	Hypertensive nephropathy	49 (55.68%)
	Diabetic nephropathy	16 (18.18%)
	Chronic glomerulonephritis	9 (10.22%)
	Nephrolithiasis	6 (6.8%)
	Polycystic kidney disease	5 (5.6%)
	Unknown	3 (3.4%)
Creatinine (mg/dL)		1.0 ± 0.4
Ca (mg/dL)		9.5 ± 0.4
P (mg/dL)		4.1 ± 0.5
PTH (pg/mL)		88 ± 62
25(OH)VitD3 (ng/mL)		19.5 ± 10
eGFR (mL/min/1.73 m^2^)		63.68 ± 19.35
FGF-23 (pg/mL)		71.2 ± 34.6
BMI (kg/m^2^)		28.15 ± 5.95
CA-IMT (mm)		0.8 ± 0.3
RRI		0.65 ± 0.06

**Table 2:** Factors associated with FGF23 in correlation analysis

Parameters	r	p
Duration of dialysis before transplantation (month)	0.22	<0.05
Ca (mg/dL)	0.54	< 0.05
P (mg/dL)	0.13	0.37
PTH (pg/mL)	0.12	0.42
eGFR (mL/min/1.73 m^2^)	-0.21	< 0.05
CA-IMT (mm)	0.18	< 0.05
RRI	0.30	< 0.05

In the multivariate analysis adjusted for age and gender, eGFR (β =-0.217, p=0.048), CA-IMT (β =0.318, p=0.009) and RRI (β =0.246, p=0.019) parameters were statistically significant, while the remaining parameters were not statistically significant (Table 3).

**Table 3:** Factors associated with FGF23 in multivariate analysis adjusted for age and gender

Parameters	β	Standard Deviation	p
Age (years)	0.085	0.007	0.646
Male Gender	0.07	0.13	0.637
Duration after transplantation (months)	-0.163	0.001	0.295
Cyclosporine -MMF/MNa-steroid	-0.261	0.46	0.571
Tacrolimus-MMF/MNa-steroid	-0.476	0.44	0.292
Ca (mg/dL)	0.071	0.001	0.532
eGFR (mL/min/1.73 m^2^)	-0.217	0.003	0.048*
CA-IMT (mm)	0.318	0.030	0.009*
RRI	0.246	0.030	0.019*

The study patients were divided into two groups according to the mean FGF23 (71.2 ± 34.6 pg/mL) level. Mean FGF23 level lower than 71 pg/mL were included in group 1 (n=42), the others (mean FGF23 level higher than 71 pg/mL) were included in group 2 (n=46). In the group analysis, eGFR was significantly lower in the patients in group 2 than in the patients in group 1 (60±12 vs.72±18, p<0.05). Again, in the group analysis, Ca (9.6 ± 0.3 vs. 8.8 ±0,2, p< 0.05), CA-IMT (0.9±0.2, vs. 0.6±0.3, p< 0.05) and RRI (0.69 ± 0.04 vs. 0.60±0.01, p< 0.05) were significantly higher in the patients in group 2 than the patients in group 1 (Table 4). 

**Table 4:** Comparison of patient characteristics according to mean FGF23 level

Parameters	Group 1 FGF23 <71pg/mL (n=42)	Group 2 FGF23 ≥ 71pg/mL (n=46)	p
Ca (mg/dL)	8.8 ± 0.2	9.6 ± 0.3	< 0.05
eGFR (mL/min/1.73 m^2^)	72 ± 18	60 ± 12	< 0.05
CA-IMT (mm)	0.6 ± 0.3	0.9 ± 0.2	< 0.05
RRI	0.60 ± 0.01	0.69 ± 0.04	< 0.05

According to literature, sub-clinical atherosclerosis is defined as CA-IMT ≥ 0.9 mm. In nearly one-third of all subjects (n=28), CA-IMT were ≥0.9 mm and in two-thirds of subjects (n=60) CA-IMT were < 0.9 mm [20]. The FGF23 levels in patients with sub-clinical atherosclerosis were higher than those with CA-IMT < 0.9 mm patients (85 pg/mL, vs. 69 pg/mL; p < 0.05). Also, the mean RRI levels in patients with CA-IMT ≥ 0.9 mm were higher than patients with CA-IMT < 0.9 mm (0.73, vs. 0.63; p < 0. 05).

## Discussion

CVDs are still the cause of more than 50% of deaths in renal transplant recipients with functional grafts [21]. Renal transplant improves renal function and abnormal mineral metabolism; however, the development of atherosclerosis has been found to be more common in renal transplant recipients with a dialysis duration more than four years [21]. Related to dialysis duration before transplantation, there is a strong association between the presence of pre-transplant atherosclerosis and post-transplant progression of atherosclerosis [22].

FGF23 is a hormone that is mostly synthesized by osteocytes and regulates phosphate metabolism. It induces phosphaturia by suppressing Na/P co-transporters in the proximal tubule and reduced calcitriol levels [18]. FGF23 levels are associated with arterial stiffness and vascular and valve calcification, which may help us explain its harmful effects on the cardiovascular system [13]. In our study, there was a significant correlation with FGF-23 level in renal transplant recipients with duration of dialysis before transplantation, higher calcium levels, low eGFR and higher RRI. Patients included in the present analysis were dialyzed for a minimum of 26 months (median 42 months) before renal transplantation. At the time of transplantation; dialysis period, plus the period of CKD prior to dialysis, cardiovascular changes would have been developing due to various metabolism disorders associated with chronic renal failure. Particular attention should be paid to the period of CKD prior to dialysis, and efforts should be made to minimize cardiovascular risks during this period. Patients should be followed up closely during this period and especially patients with live donors should be evaluated quickly for preemptive transplantation. This approach will minimize patients' CKD process and uremic toxin exposure. Thus, after the transplantation, patients could have more benefit for cardivascular protection and graft functions.

In renal transplant recipients with good graft functions (eGFR> 30-45 ml/min), high PTH levels are detected in the first year [23]. Since the mean duration after transplantation was 61.2 ± 22.5 months in our study, PTH levels were 88 ± 62 and there was no significant correlation in the group with a FGF-23 level of >71. High FGF23 level has been found to be more common in renal transplant recipients with a long dialysis duration in our study population. And also in our analysis, serum FGF23 levels were elevated in most patients but particularly in those with a high CA-IMT score. Similar to our study, a study by Balci M et al. on 200 hemodialysis (HD) patients found a significant positive correlation between plasma FGF23 levels and CA-IMT [24]. In contrast, the study by Ashikaga E et al. on 196 HD patients showed a negative correlation between FGF23 levels and CA-IMT [25]. The study by Ashikaga E et al. included hemodialysis patients and not renal transplant patients, and their patients had higher phosphorus levels and lower calcium levels compared to our patients [25]. In addition to this, in the study by Ashikaga E et al., the fact that FGF-23 levels was not a marker of atherosclerosis despite the high level of phosphorus may be due to better nutritional status in hemodialysis patients with high phosphorus level. Although our patients had similar CaXP levels, the longer period of exposure to elevated CaXP due to the high-dose immunosuppressive therapy used, higher calcium level, low vitamin D, and being an older patient group was thought to be more effective in atherosclerosis. Previous studies have demonstrated that high serum calcium levels and calcium phosphate product might be considered as a ‘vascular toxin’ and closely associated with atherosclerosis [26,27]. Post-transplantation persisting hypercalcemia and hypophosphatemia have been reported to result in complications such as vascular calcification and loss of graft function [28-29]. Normally, FGF23 levels should increase as vitamin D level decreases; however, a significant correlation might not have been found since it was low in all patients and the mean duration after transplantation was longer. Moreover, the mean BMI value of the renal transplant recipients included in our study was 28 kg/m2 and the patients had obesity, which is one of the causes of vitamin D3 deficiency.

Our study has some limitations that negatively affected the results. First, our study was a single-center study and conducted with a small sample size. Second, the temporal effects of the correlation between FGF23 graft function and CV events could not be investigated in renal transplant recipients since it was a cross-sectional study. Third, the characteristics of the patients were not compared with that of healthy individuals and CKD patients with similar age and GFR values. Because of the limited number of studies on this subject, further multi-center studies with a large sample size are needed.

## Conclusions

FGF23 can be considered as a descriptive biomarker for cardiovascular prognosis and graft function for patients with kidney transplantation. Preemptive kidney transplantion should be encouraged for all CKD patients with live donors. CA-IMT measurement should be used more frequently to assess cardiovascular risk parameters for these patients.
